# A non-canonical RNAi pathway controls virulence and genome stability in Mucorales

**DOI:** 10.1371/journal.pgen.1008611

**Published:** 2020-07-13

**Authors:** Carlos Pérez-Arques, María Isabel Navarro-Mendoza, Laura Murcia, Eusebio Navarro, Victoriano Garre, Francisco Esteban Nicolás

**Affiliations:** Department of Genetics and Microbiology, Faculty of Biology, University of Murcia, Murcia, Spain; Stowers Institute for Medical Research, UNITED STATES

## Abstract

Epimutations in fungal pathogens are emerging as novel phenomena that could explain the fast-developing resistance to antifungal drugs and other stresses. These epimutations are generated by RNA interference (RNAi) mechanisms that transiently silence specific genes to overcome stressful stimuli. The early-diverging fungus *Mucor circinelloides* exercises a fine control over two interacting RNAi pathways to produce epimutants: the canonical RNAi pathway and a new RNAi degradative pathway. The latter is considered a non-canonical RNAi pathway (NCRIP) because it relies on RNA-dependent RNA polymerases (RdRPs) and a novel ribonuclease III-like named R3B2 to degrade target transcripts. Here in this work, we uncovered the role of NCRIP in regulating virulence processes and transposon movements through key components of the pathway, RdRP1 and R3B2. Mutants in these genes are unable to launch a proper virulence response to macrophage phagocytosis, resulting in a decreased virulence potential. The transcriptomic profile of *rdrp1*Δ and *r3b2*Δ mutants revealed a pre-exposure adaptation to the stressful phagosomal environment even when the strains are not confronted by macrophages. These results suggest that NCRIP represses key targets during regular growth and releases its control when a stressful environment challenges the fungus. NCRIP interacts with the RNAi canonical core to protect genome stability by controlling the expression of centromeric retrotransposable elements. In the absence of NCRIP, these retrotransposons are robustly repressed by the canonical RNAi machinery; thus, supporting the antagonistic role of NCRIP in containing the epimutational pathway. Both interacting RNAi pathways might be essential to govern host-pathogen interactions through transient adaptations, contributing to the unique traits of the emerging infection mucormycosis.

## Author summary

Mucormycosis is an emergent and lethal infectious disease caused by Mucorales, a fungal group resistant to most antifungal drugs. *Mucor circinelloides*, a genetic model to characterize this infection, can develop drug resistance via RNAi epimutations. This epimutational RNAi mechanism interacts with a novel non-canonical RNAi pathway (NCRIP), where the ribonuclease III-like R3B2 and the RNA-dependent RNA polymerase RdRP1 are essential. The analysis of the transcriptomic response to macrophage phagocytosis in *rdrp1*Δ and *r3b2*Δ mutants revealed that NCRIP might control virulence in *M*. *circinelloides*. These mutants showed constitutive activation of the response to phagocytosis and a reduction in virulence in a mouse model, probably caused by a disorganized execution of the genetic program to overcome host defense mechanisms. The antagonistic role of the NCRIP and the RNAi canonical core is evident during post-transcriptional regulation of centromeric retrotransposons. These retrotransposons are silenced by the canonical RNAi pathway, but this regulation is restrained by NCRIP, proven by an overproduction of small RNAs targeting these loci in NCRIP mutants. These new insights into the initial phase of mucormycosis and transposable element regulation point to NCRIP as a crucial genetic regulator of pathogenesis-related molecular processes that could serve as pharmacological targets.

## Introduction

Mucorales are a group of ancient fungi that are emerging as a new source of pathogens causing the fungal infection mucormycosis. This infectious disease is increasing the focus of recent studies due to its high mortality rates, which can reach up to 90% in cases of disseminated infection [1,2]. The elevated mortality rate is a direct connection to a lack of effective antifungal treatments, a consequence of the unusual resistance observed in these fungi. In this regard, a novel RNAi-dependent epimutational mechanism of drug resistance has been described in *M*. *circinelloides* [3]. In this mechanism, *M*. *circinelloides* generates strains resistant to the antifungal drug FK506 after only four days of exposure. The mechanism behind this rapid adaptation relies on the specific silencing of the *fkbA* gene and its encoded FKBP12 protein, which is the target of FK506. Thus, in the absence of FKBP12 due to *fkbA* silencing, the drug FK506 is unable to hinder the mycelial growth of *M*. *circinelloides*, generating transient resistant strains that arise due to selective pressure. The epimutational drug resistance in *M*. *circinelloides* is becoming clinically relevant because epimutants can emerge upon exposure to other antifungal drugs [4], and they exhibit organ-specific stability during *in vivo* infection [5].

The RNAi pathway involved in this epimutation-based drug resistance depends on the canonical components of the RNAi machinery, which are broadly characterized in *M*. *circinelloides* [6]. First, RNA dependent RNA polymerases (RdRPs) generate double-stranded RNA (dsRNA). Later, dsRNA is processed by RNase III Dicer enzymes to generate small RNAs (sRNAs). Then, the third element of the RNAi canonical core, the Argonaute protein (Ago), uses the sRNAs to conduct homology-dependent repression of the target sequences [7]. Besides drug-resistance, the canonical core elements participate in RNAi-based defensive pathways protecting genomic integrity against invasive nucleic acids and transposable elements, as well as in other RNAi pathways involved in the endogenous regulation of target mRNAs [8].

Although epimutants can arise in wild-type strains, the phenomenon is enhanced by mutations in key genes of an RdRP-dependent Dicer-independent degradation mechanism for endogenous mRNA [3,9]. This could mean that either this novel RNAi pathway directly represses the epimutation machinery or that it competes for the same target mRNAs. This degradation mechanism is considered a non-canonical RNA interference pathway (called NCRIP) because it does not share the canonical core RNAi machinery. Indeed, mutational analyses showed that only RdRP enzymes, but neither Dicer nor Argonaute, participate in the NCRIP pathway [10]. The cleaving activity required to degrade target mRNAs relies on a new RNase III-like protein named R3B2, which plays the primary RNase role in the NCRIP pathway. The unique role of RdRPs (RdRP1, RdRP2, and RdRP3) in RNA degradation suggests that the NCRIP mechanism represents a first evolutionary link connecting mRNA degradation and post-transcriptional gene silencing [9].

The role of NCRIP in regulating the RNAi-dependent epimutational mechanism emphasizes the intricate network of interactions among RNAi pathways in fungi. However, the actual functional role of NCRIP in cellular processes and the importance of its regulatory effects on fungal physiology are still unknown. The large number of predicted genes that might be regulated by NCRIP suggested a pleiotropic role in fungal physiology, controlling several and diverse processes. Indeed, phenotypic analysis of mutants lacking the NCRIP pathway revealed two prominent phenotypes associated with the lack of NCRIP: *in vitro* oxidative stress resistance and reduced production of zygospores during sexual development [10].

RNAi-related mechanisms are important for the maintenance of genome stability and transposon movement in other fungal pathogens such as *Cryptococcus neoformans* [11]. In this basidiomycete, the canonical RNAi machinery plays a protective role by silencing transposable elements during mating, ensuring the genomic integrity of the progeny. A recent study in *M*. *circinelloides* also found an essential role for the canonical RNAi core in silencing repetitive pericentric transposable elements [12]. Interestingly, analysis of genome-wide sRNA content in epimutants that were deficient in NCRIP revealed an alteration of sRNA levels derived from transposable elements [4]. These studies reinforce the hypothesis of an inhibitory function of NCRIP over the canonical pathway during the production of epimutants. Thus, NCRIP could have a role in maintaining genome integrity through its competitive regulation of the canonical RNAi in the control of transposable elements. Moreover, the resistance to oxidative stress observed *in vitro* in NCRIP deficient mutants [10] could play a specific role for survival in stressful environments, such as those related to the host-pathogen interaction. NCRIP may also be involved in pathogenesis given the high frequency of drug-resistant epimutants in mutants of this pathway, suggesting that this regulatory mechanism could be linked to virulence in *M*. *circinelloides*.

Here, we show a detailed functional analysis of the NCRIP pathway, addressing the functional roles that it might play in fungal biology and pathogenesis. Consequently, we studied the complex network of genes regulated by NCRIP during saprophytic growth and macrophage phagocytosis. This study identifies the complete profile of genes and functional categories regulated by NCRIP in both conditions. Interestingly, most of the fungal genes regulated by phagocytosis are under the control of NCRIP, indicating that this RNAi-based mechanism is a master regulator of the response of the pathogen to phagocytosis.

## Results

### NCRIP preferentially regulates functional processes during non-stressful conditions

**Fig 1:**
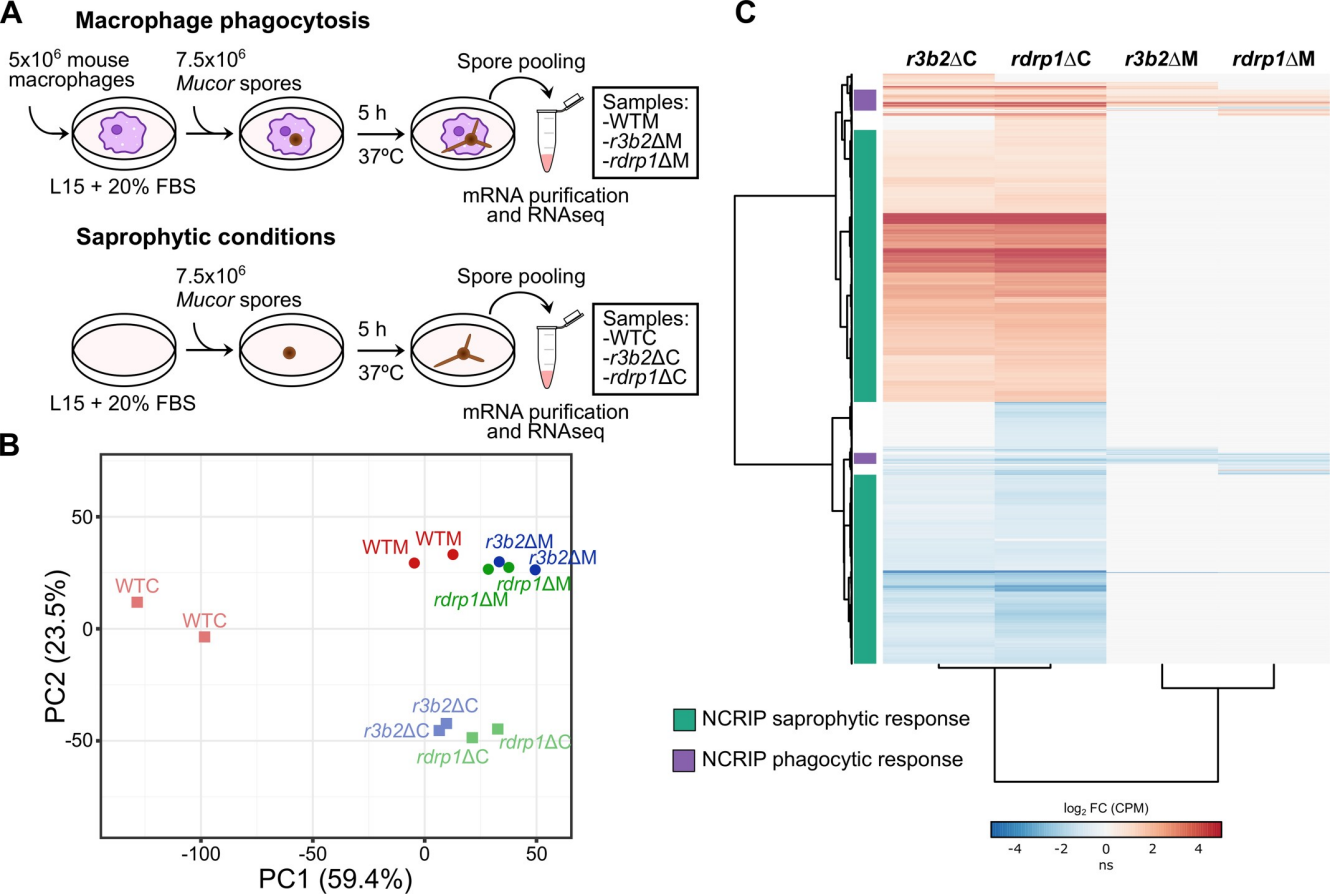
NCRIP regulates a vast gene network via the cooperation of R3B2 and RdRP1. **(A)** Diagram of the experimental design followed to perform macrophage phagocytosis and saprophytic assays for RNA sequencing. **(B)** Principal component (PC) analysis biplot of transcript abundances (measured as counts per million [CPM], mean CPM > 1.0), showing the variability across the color- and shape-coded samples. **(C)** Heatmap of significant differentially expressed genes (DEGs, log_2_ FC ≥ |1.0|, FDR ≤ 0.05) in the depicted NCRIP mutant strains and conditions compared to the wild-type strain in the same condition as a log_2_ ratio. Differential expression values and experimental samples are clustered by similarity. All DEGs responding exclusively in the saprophytic condition in both NCRIP mutants are clustered in green. DEGs in at least one NCRIP mutant responding to macrophage phagocytosis are depicted in purple. Differential expression values (in log_2_ CPM) are color-coded in red for upregulation and blue for downregulation (ns stands for non-significant changes).

We performed a transcriptomic analysis of the gene expression profiles obtained from high-throughput sequencing of mRNA (RNA-seq) from spores of the wild-type strain and mutants lacking NCRIP activity (*r3b2*Δ or *rdrp1*Δ). The spores were single-cultured in rich medium L15 for saprophytic conditions, and co-cultured with the J774A.1 cell-line of mouse macrophages (1.5:1 spore‒macrophage ratio) for 5 hours to ensure that most of the spores were phagocytosed (Fig 1A). These macrophage phagocytosis assays represent a close *in vitro* environmental approach to a clinical *in vivo* context in which the germinating spores must rapidly overcome oxidative stress to escape from the innate immune response.

Messenger RNA was isolated and deep sequenced to analyze the transcriptional response of the wild-type control samples with or without macrophages (Fig 1B, WTM or WTC, respectively), and the two mutant samples, with macrophages (Fig 1B, *r3b2*ΔM and *rdrp1*ΔM) or without (Fig 1B, *r3b2*ΔC and *rdrp1*ΔC). We performed a principal component analysis of the expression values for all genes (mean CPM > 1.0 per gene in all conditions) to further study the variability among the samples (Fig 1B). This analysis revealed that the NCRIP mutant strains, *r3b2*Δ and *rdrp1*Δ, clustered closely together and had a distinct transcriptomic profile compared to the wild-type strain growing in saprophytic conditions. However, the expression profile of all strains formed a closer cluster and showed a more similar profile during macrophage phagocytosis. To identify these changes in gene expression, the genetic profiles of the two mutants were compared to the wild-type strain in the presence or absence of macrophages (S1 Data and S1 Fig). A threshold of a corrected p-value of less than or equal to 0.05 (False Discovery Rate [FDR] ≤ 0.05) and a log_2_ fold change greater than or equal to 1.0 (log_2_ FC ≥ |1.0|) was selected to consider differentially expressed genes (DEGs). In addition, we analyzed the expression of three housekeeping genes to ensure a correct normalization among samples (S1 Fig). The deletion of either *r3b2* or *rdrp1* caused a profound variation in the mRNA profiles of *M*. *circinelloides*, especially when the fungus grew without macrophages (Table 1). Under these saprophytic conditions, most DEGs trend towards upregulation in the mutants, as expected by the lack of the direct repressive activity of NCRIP. Downregulated DEGs in the mutant spores comprise NCRIP secondary targets, suggesting that NCRIP represses key regulators of a vast network of these secondary targets in the wild-type strain (Table 1 and S1 Fig).

**Table 1:** Differentially expressed genes in NCRIP mutant strains compared with the wild-type strain.

Culture conditions	Strain	Upregulated genes^1^		Downregulated genes^2^	
		#	Average log_2_ FC^3^	#	Average log_2_ FC^3^
L15 5h 37°C	*r3b2*Δ	1637	2.33 ± 1.32	870	-1.52 ± 0.68
	*rdrp1*Δ	1869	2.37 ± 1.38	1481	-1.65 ± 0.72
L15 5h 37°C + Mφ	*r3b2*Δ	84	1.78 ± 1.46	94	-1.66 ± 0.78
	*rdrp1*Δ	78	1.84 ± 1.50	107	-1.57 ± 0.67

^1^FDR ≤ 0.05, log_2_ FC ≥ 1.0, average CPM > 1.0

^2^FDR ≤ 0.05, log_2_ FC ≤ -1.0, average CPM > 1.0

^3^Average of all log_2_ fold change values ± standard deviation

Subsequently, we searched for shared DEGs in both NCRIP mutants compared to their wild-type control, clustering them in a heatmap of differential expression values (Fig 1C). The analysis revealed a total of 3187 genes (>25% of the genome) regulated by both R3B2 and RdRP1 under any condition, highlighting the regulatory function of the NCRIP in *M*. *circinelloides*. We identified two distinct groups of genes comparing the NCRIP mutant samples with their proper wild-type control: a group that encompasses 2333 DEGs shared in *r3b2* and *rdrp1* mutants in saprophytic conditions (1542 upregulated and 791 downregulated, NCRIP saprophytic response), and a second group comprised of 101 DEGs (49 upregulated and 52 downregulated, NCRIP phagocytic response) in phagocytic conditions (Fig 1C). These higher differences in saprophytic conditions and the low number of DEGs in the presence of macrophages agree with the results observed in the principal component analysis (Fig 1B). Indeed, the vast majority of the DEGs in both responses are shared by the two NCRIP mutants, confirming the role of R3B2 and RdRP1 as key proteins in the same silencing mechanism.

**Fig 2:**
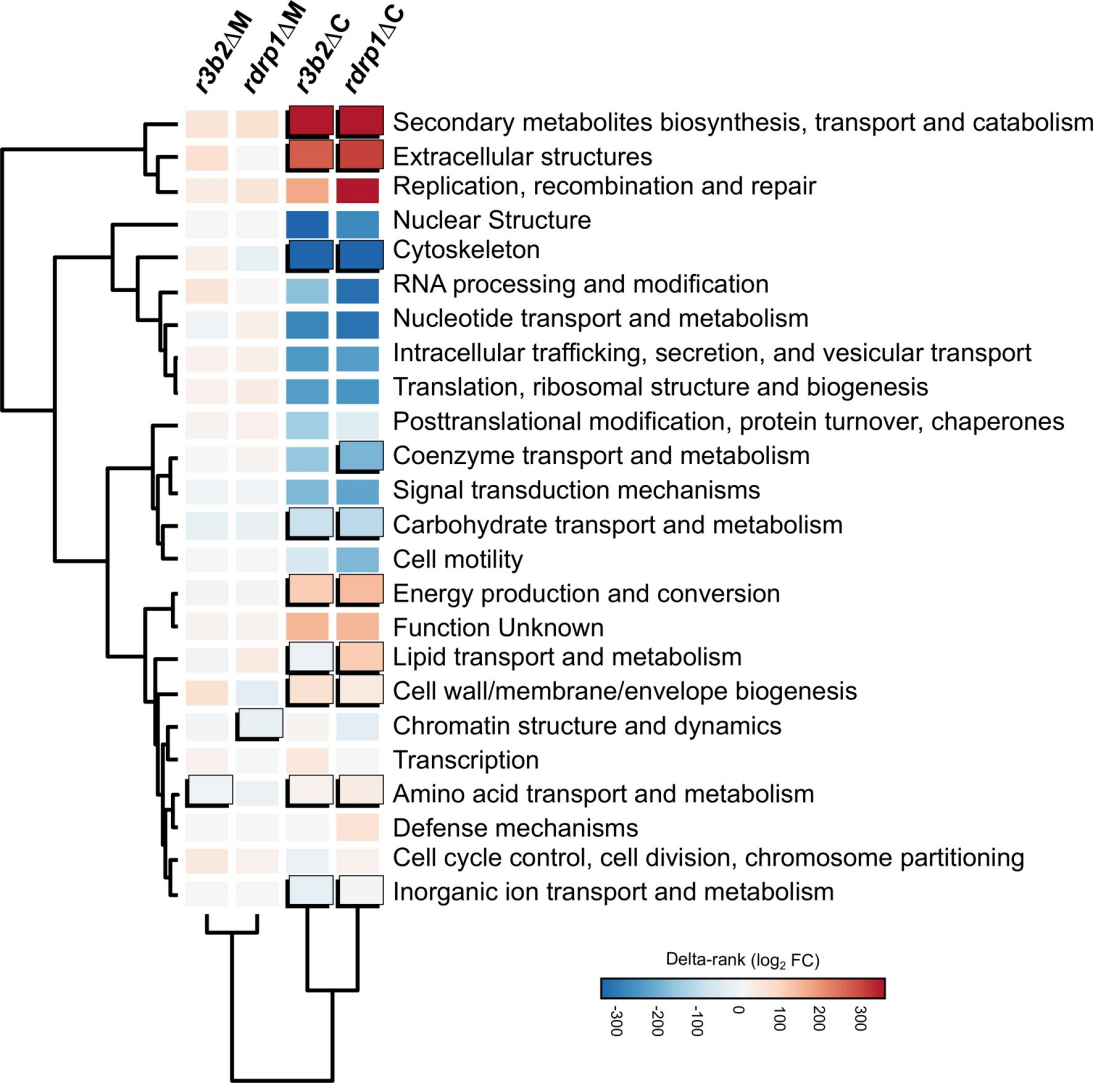
NCRIP regulates key functional categories involved in saprophytic growth. Enrichment analysis of DEGs in each Eukaryotic Orthologous Groups (KOG) class. Significant enrichments (Fisher’s exact test, P ≤ 0.05) in a given mutant strain and condition compared to the wild-type strain are shown as uplifted rectangles. A measure of up- (red) or downregulation (blue) of each KOG class is represented as a colored scale of delta-rank values (the difference between the mean rank differential expression value of all genes in each KOG class and the mean rank differential expression value of all other genes). KOG classes and experimental conditions (mutant strains and presence/absence of macrophages) are clustered according to the similarity of their delta rank values.

An enrichment analysis of Eukaryotic Orthologous Groups (KOG) terms surveyed the possible cellular processes controlled by the NCRIP machinery in saprophytic growth without any challenge. Under these non-stressful conditions, we found an enrichment in processes related to the production of extracellular structures and secondary metabolites, the remodeling of energy, inorganic ion, amino acid, lipid, and carbohydrate metabolic pathways, and by contrast an overall reduction in cytoskeletal processes (Fig 2). None of the enriched KOG classes were shared by the two mutants during phagocytosis, possibly because the gene set was too small to produce a significant result. Instead, *r3b2*Δ and *rdrp1*Δ mutants showed independent roles in amino acid transport and metabolism, and chromatin structure and dynamics, respectively, suggesting that these genes perform specific roles required during phagocytosis that are not controlled by NCRIP. Altogether, our transcriptomic analysis showed that most of NCRIP target genes were differentially expressed during saprophytic growth and enriched in essential functions that govern metabolism and germination processes. These conclusions indicate that NCRIP represses its target genes under non-stressful conditions and releases its control when the spores are phagocytosed.

### NCRIP repressed the genetic response to phagocytosis during non-stressful conditions

**Fig 3:**
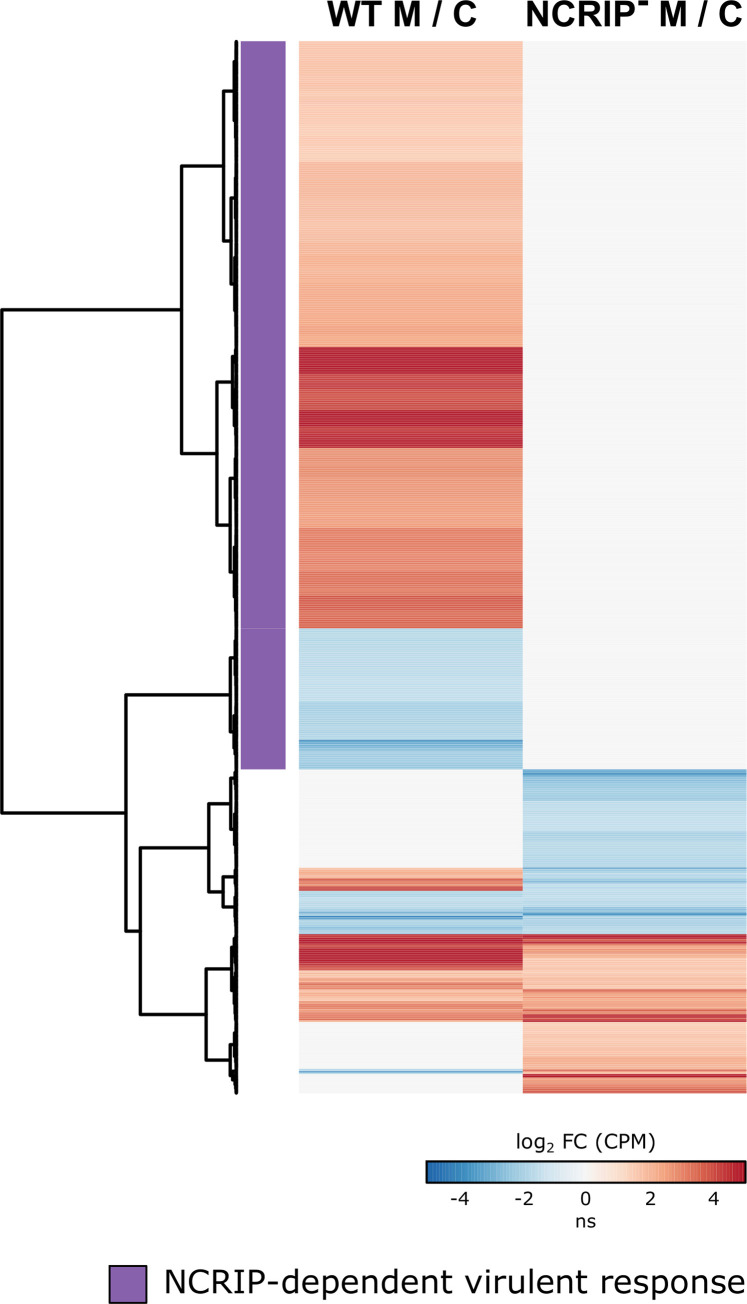
*M*. *circinelloides* coordinates an NCRIP-dependent virulent response to phagocytosis. Heatmap of DEGs (log_2_ FC ≥ |1.5|, FDR ≤ 0.05) in the wild-type strain and the NCRIP mutant strains during phagocytosis compared to their saprophytic control conditions as a log_2_ ratio. Red and blue represent up- and downregulated genes, respectively (ns stands for non-significant changes). Differential expression values (in log_2_ CPM) are clustered by similarity. DEGs responding to macrophage phagocytosis in the wild-type strain but not in the NCRIP mutants are clustered together and colored in purple, constituting the NCRIP-dependent virulent response.

Previous studies revealed an intricate network of genes activated in response to phagocytosis in a virulent strain, which is essential for the pathogenic potential of Mucorales [13]. Considering the large number of genes and functional processes regulated by NCRIP, we postulated that lacking this silencing pathway could affect *M*. *circinelloides* response to phagocytosis. To address this hypothesis, we analyzed the DEGs detected in response to phagocytosis in the wild-type strain and in the NCRIP mutants. Because the transcriptomic profiles of both *rdrp1*Δ or *r3b2*Δ strains are clearly similar and both proteins are involved in the NCRIP pathway, they were analyzed as biological replicates (S2 Fig and S2 Data). We identified a wide response to phagocytosis in the wild-type strain that did not appear in the NCRIP mutants (Fig 3), suggesting that these genes might be controlled by this mechanism. The absence of this virulent response in the NCRIP mutants is explained by the lack of the silencing mechanism, making the spore unable to launch a wild-type response to phagocytosis. Thus, a cluster of 1162 DEGs (937 up- and 225 downregulated) responding to phagocytosis in the wild-type strain but not in the NCRIP mutants correspond to an NCRIP-dependent virulent response (Fig 3). Two alternative possibilities could explain this response: a functional NCRIP is required either to induce the response to phagocytosis or to repress this response under non-stressful conditions.

**Fig 4:**
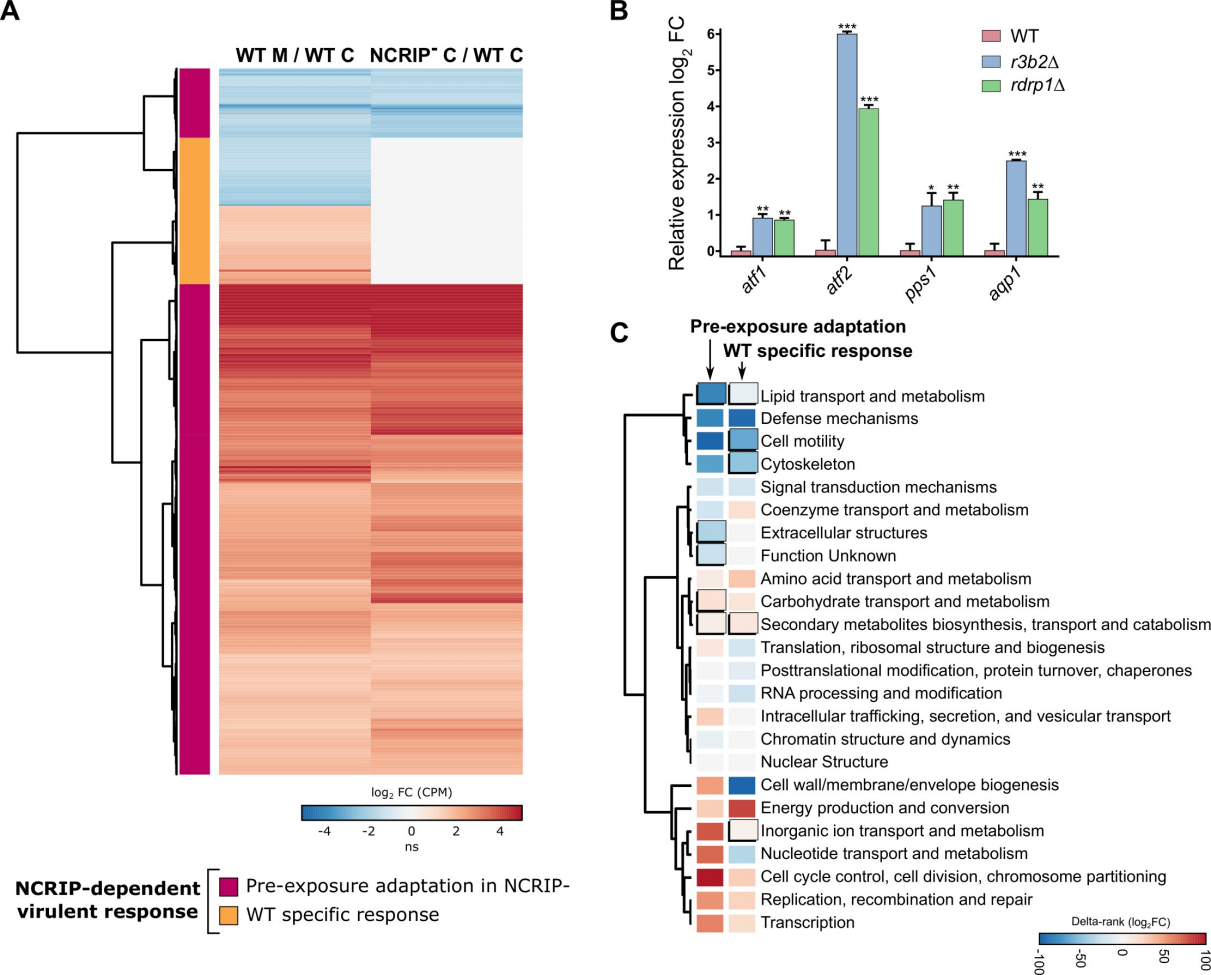
NCRIP controls the response to macrophage phagocytosis by inhibiting its targets under non-stressful conditions. **(A)** The NCRIP-dependent virulent response is depicted in a heatmap. Genes are clustered by similarity to compare the response to phagocytosis in the wild-type strain and the response of the NCRIP mutants in saprophytic conditions. This comparison reveals a pre-exposure adaptation of the NCRIP mutants (magenta cluster), showing similar expression values than the wild-type response to phagocytosis. Exclusive genes of the wild-type response are clustered together (orange cluster). Differential expression values (in log_2_ CPM) are color-coded to depict the degree of upregulation (red) or downregulation (blue) in each condition (ns stands for non-significant changes). **(B)** Bar plot of *atf1*, *atf2*, *pps1* and *aqp1* expression differences in *r3b2*Δ, and *rdrp1*Δ mutant strains compared with the wild-type strain in non-stressful conditions, i.e., incubation in cell-culture medium without macrophages for 5 hours. Log_2_ fold-change differential expression levels were quantified by RT-qPCR and normalized using *rRNA* 18S as an internal control. Error bars correspond to the SD of technical triplicates and significant differences are denoted by asterisks (* for P ≤ 0.05, ** for P ≤ 0.005, and *** for P < 0.0001 in an unpaired t-test). **(C)** KOG class enrichment analysis of the NCRIP-dependent virulent response during macrophage interaction is grouped in pre-exposure adaptation in NCRIP mutants and wild-type specific responses. Significant enrichments (Fisher’s exact test, P ≤ 0.05) are shown as uplifted rectangles. A measure of up- (red) or downregulation (blue) of each KOG class is represented as a colored scale of delta-rank values (the difference between the mean rank differential expression value of all genes in a particular KOG class and the mean rank differential expression value of all other genes). KOG classes are clustered according to the similarity of their delta rank values.

Our previous results suggest that NCRIP acts preferentially under saprophytic conditions, regulating a large number of genes (Fig 1C). Therefore, we further compared the NCRIP-dependent virulent response with the NCRIP saprophytic response to clarify the role of this silencing mechanism during phagocytosis (Fig 4A). Surprisingly, the differential expression profile in the wild-type strain responding to phagocytosis was almost identical to that found in the NCRIP mutants in saprophytic conditions (Fig 4A). This analysis showed a group of genes responding to both macrophage-mediated phagocytosis in the wild-type strain and the lack of NCRIP activity (cluster purple in Fig 4A). Lacking NCRIP proteins provokes a similar response as the one observed when the wild-type spores are phagocytosed, which can be explained as a pre-exposure adaptation of the NCRIP mutants in saprophytic conditions. This response is comprised by a group of activated genes that may correspond to primary target genes repressed by NCRIP (Fig 4A, upregulated genes in red), suggesting a negative regulation of NCRIP in the absence of macrophages that is released upon phagocytosis in the wild-type strain. A second group consists of genes repressed both by the presence of macrophages in the wild-type strain and the lack of NCRIP activity (Fig 4A, downregulated genes in blue). These genes could be acting as secondary targets of the primary gene set. In contrast, a cluster of genes responding in the wild-type strain to phagocytosis is not preactivated in the NCRIP mutants (Fig 4A, orange cluster). Therefore, this is a wild-type specific response to phagocytosis, controlled by the NCRIP exclusively during phagocytosis.

Previous studies identified gene expression profiles during the phagocytosis of *M*. *circinelloides* wild-type spores [13]. Those profiles were validated by quantitative RT-PCR using the following representative marker genes: *atf1*, *atf2*, *pps1*, and *aqp1*. These marker genes showed a significant induction during macrophage phagocytosis and are essential for this fungal pathogen to survive and cause infection. Our transcriptomic analysis found that all of these marker genes were also controlled by NCRIP during saprophytic growth (S2 Data), and thus, they were employed here to validate the transcriptional pre-exposure adaptation observed in the NCRIP mutants, *rdrp1*Δ and *r3b2*Δ, without macrophages (Fig 4B). We found that the four marker genes showed a significant induction in the two mutants in saprophytic conditions, similar to the previously reported increased expression observed in the wild type during phagocytosis [13]. These results validate the transcriptional profiles observed in the pre-exposure adaptative response, indicating that NCRIP controls the response to phagocytosis by repressing its targets during saprophytic conditions.

Functional enrichment analyses of the NCRIP-dependent virulent response were performed to further understand the biological processes involved in the pre-exposure adaptative and WT-specific responses (Fig 4C). We observed a clear alteration that affects the metabolism and transport of both carbohydrates, lipids, and secondary metabolites and the production of extracellular structures in the pre-exposure adaptive response. While most of these metabolic functions were also enriched in the WT-specific response, we found exclusive functions related to cell motility, cytoskeleton, and inorganic ion transport and metabolism, which could be important for pathogenesis.

### Lack of NCRIP confers higher tolerance to oxidative stress but decreases virulence

**Fig 5:**
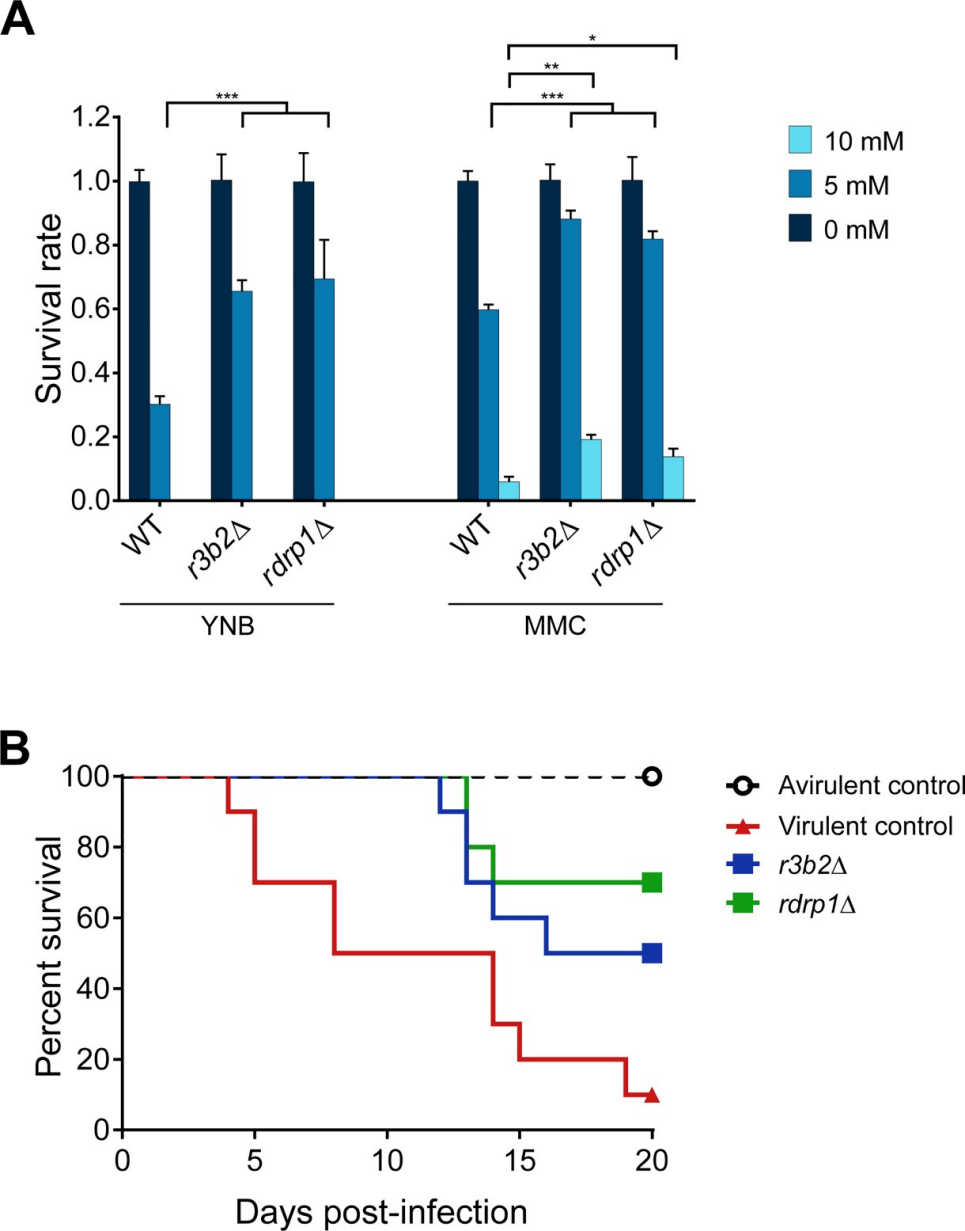
NCRIP is involved in oxidative stress tolerance and mucormycosis. **(A)** A bar plot showing the survival rates of the NCRIP mutants (*r3b2*Δ and *rdrp1*Δ) compared to a wild-type strain under oxidative stress. Survival assays were performed in two different minimal media (YNB and MMC) supplemented with two different H_2_O_2_ concentrations: 5 mM and 10 mM. Error bars correspond to the SD of technical triplicates and significant differences in survival rates were denoted by asterisks (* for P ≤ 0.05, ** for P ≤ 0.005, and *** for P < 0.0001 in a two-way ANOVA with Tukey’s multiple comparison test). **(B)** The virulence of *r3b2*Δ and *rdrp1*Δ mutant strains was assessed in a survival assay using immunosuppressed mice as a mucormycosis model. Groups of ten mice were infected intravenously with 1x10^6^ spores from each strain (color-coded). Survival rates were statistically analyzed for significant differences (P ≤ 0.05 in a Mantel-Cox test) compared with a virulent control strain (R7B). NRRL3631 was used as an avirulent mock control of infection.

The metabolic changes observed in the pre-exposure adaptation could be linked to the germination process inside the phagosome. The harsh phagosomal environment might also be responsible for the induction of genes related to the biosynthesis, transport, and catabolism of secondary metabolites and extracellular structures to defend the fungus from an oxidative challenge. To test this hypothesis, mutants *r3b2*Δ and *rdrp1*Δ were used to perform a germination assay mimicking the oxidative environment of the phagosome (Fig 5A). Both NCRIP mutants showed significant higher survival rates confronting oxidative stress compared to the wild-type strain at moderate and high H_2_O_2_ concentrations (5 mM and 10 mM, respectively). The lack of NCRIP activity provokes a pre-exposure adaptation to stress, resulting in a higher tolerance to oxidative stress.

Virulence is a complex trait that depends on multiple genes and is controlled by different biological processes [14]. The role of NCRIP in the regulation of the response to phagocytosis might be important to launch a proper invasive infection. Because the NCRIP-dependent virulent response is enriched in functions related to pathogenesis, we postulated that the lack of NCRIP might affect the virulent potential of *M*. *circinelloides* spores. Consequently, the *r3b2*Δ and *rdrp1*Δ mutants were used to perform survival assays in an immunosuppressed mouse model, previously validated as a host model for infections with *M*. *circinelloides* [15]. The survival rates were compared to those of mice injected with the wild-type virulent strain R7B and the NRRL3631 strain, a natural soil isolate that served as an avirulent mock control [16]. The results of these assays showed a significant reduction in virulence of the two mutant strains (Log-rank Mantel-Cox test, p = 0.0061 in *rdrp1*Δ vs. R7B; p = 0.040 in *r3b2*Δ vs. R7B; Fig 5B). Surprisingly, these results indicate that despite being pre-adapted to oxidative stress, the NCRIP mutants could not develop a proper virulent infection.

### NCRIP negatively regulates the protective role of the canonical RNAi pathway in the suppression of Grem-LINE1s retrotransposons

**Fig 6:**
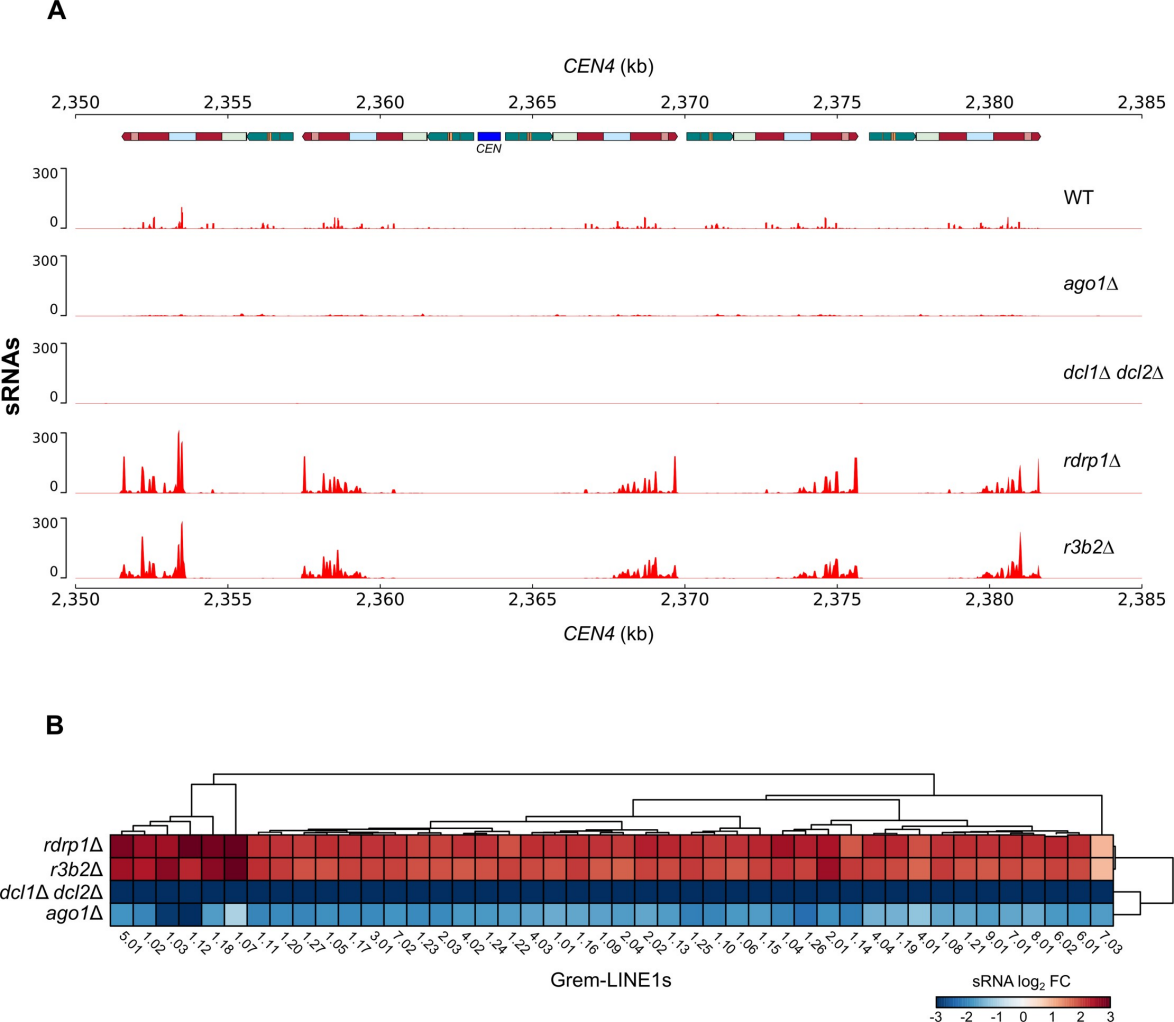
NCRIP competes with the epimutational pathway to regulate transposable elements. **(A)** A genomic view of centromeric chromatin (*CEN4*) displaying the kinetochore-binding region enrichment that marks the centromere (*CEN*, blue), annotation of transposable elements (colored blocks), and transcriptomic data of sRNAs (red) in *M*. *circinelloides* wild-type, canonical pathway (*ago1Δ*, double *dcl1Δ/dcl2Δ*) and NCRIP (*rdrp1Δ* and *r3b2Δ*) deletion mutant strains after 48 h of growth in rich medium. sRNA values are normalized to bins per million (BPM) mapped reads. **(B)** Heatmap of the differential sRNA accumulation targeting Genomic retrotransposable elements of Mucoromycotina LINE1-like (Grem-LINE1s) in the depicted RNAi mutants compared to the RNAi-proficient wild-type strain. Grem-LINE1s are numbered according to Navarro-Mendoza et al. classification [12]. Differential sRNA values (in log_2_ CPM) are normalized by the trimmed mean of M values (TMM).

The results presented above indicate that NCRIP represses genes during saprophytic growth and then releases its control upon macrophage phagocytosis, a clear challenging stimulus. However, the expression profiles analyzed did not reveal any specific pathway involved in sporulation or mating. Because mutants in the machinery of NCRIP also display defects in the production of zygospores during mating [10], we hypothesized that NCRIP might also contribute in the regulation of genes involved in other stresses such as antifungal agents [4,9] and genomic integrity stress, which could alter complex cell processes involved in mating [10]. A recent study supported this hypothesis, unveiling a direct link between the canonical RNAi pathway and the protection of genome integrity against transposable elements in *M*. *circinelloides* [12]. The pericentric regions of *M*. *circinelloides* contain a large number of L1-like retrotransposable elements of Mucoromycotina species called Grem-LINE1s, which are actively silenced by the canonical RNAi machinery. As suggested previously [9], NCRIP might regulate the RNAi canonical core during the control of these transposable elements by suppressing the epimutational pathway. In this sense, we characterized the production of sRNAs from Grem-LINE1 transcripts in the pericentric regions of the wild-type strains and the *r3b2*Δ *and rdrp1*Δ mutants (Fig 6A). The pericentric regions are almost depleted of sRNAs in the *ago1*Δ and *dcl1 dcl2*Δ mutants, whereas the wild-type strain exhibited an active production of sRNAs aligned to these loci, as previously reported [12]. Interestingly, the *r3b2*Δ *and rdrp1*Δ mutants displayed an increased production of sRNAs compared to the wild-type strain. We performed a differential sRNA production analysis to assess this silencing pattern across all Grem-LINE1s (Fig 6B and S3 Data). Also, the correlation in sRNA production across the rest of the genome and, especially, in three non-RNAi regulated housekeeping genes was examined to ensure a proper normalization among samples (S3 Fig). The over-accumulation of sRNAs (≥ 1.5 log_2_ FC) is consistent among all Grem-LINE1s in the *r3b2*Δ *and rdrp1*Δ mutants (Fig 6B), while in the canonical pathway mutants (*ago1*Δ and *dcl1 dcl2*Δ) the levels of sRNAs are drastically decreased. The production of sRNA from the Grem-LINE1s correlates inversely with their mRNA levels (S4 Fig), thus confirming the mechanism of the silencing pathway.

**Fig 7:**
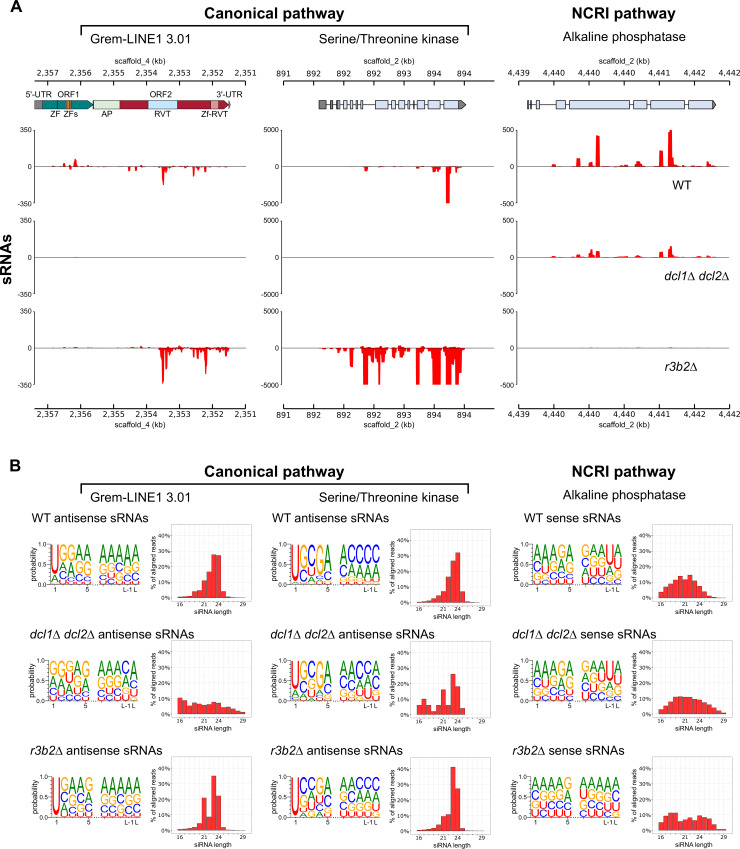
Transposable element sRNAs exhibit typical characteristics of the canonical RNAi pathway biogenesis. **(A)** Representative diagram of sRNA production in the canonical and NCRI pathways. The canonical pathway is represented in two loci: Grem-LINE1, and a Serine/Threonine kinase (JGI Muccir1_3 ID: 1455000) [17]. NCRIP is exemplified by an Alkaline phosphatase (JGI Muccir1_3 ID: 1469159) [10]. Sense (positive values) and antisense (negative values) sRNAs accumulation is shown; sRNA values are normalized to bins per million (BPM) mapped reads. Grem-LINE1 open reading frames (ORF1 and ORF2 as green and red arrows, respectively) and protein domains predicted from its coding sequence are shown as colored blocks (zf-RVT, zinc-binding in reverse transcriptase [PF13966]; RVT, reverse transcriptase [PF00078]; AP, AP endonuclease [PTHR22748]; and ZF, zinc finger [PF00098 and PF16588]). **(B)** Sequence analysis of sRNAs mapping to the three loci shown in (A), produced in a wild-type, *dcl1*Δ/*dcl2*Δ, and *r3b2Δ* mutant strains. Each sequence analysis depicts a probability logo (showing the first and last five nucleotides) and a size distribution bar plot.

The sRNAs produced by each silencing pathway, the canonical pathway and NCRIP, show specific features. The canonical pathway produces sRNAs both sense and mainly antisense to the transcript, with a defined length of 21–24 nt and uracil at the 5’ position [17]. On the contrary, NCRIP sRNAs have a strong strand bias being mostly sense, a uniform size distribution (16–29 nt), and uracil in the second-to-last position [10]. We searched for these structural features in the sRNAs sequences from Grem-LINE1s and compared them to the sRNAs produced by two control loci: one regulated by the canonical RNAi pathway, coding for a serine/threonine kinase [17]; and other by NCRIP, coding for an alkaline phosphatase [10] (Fig 7). In the wild-type strain, the sRNAs from the Grem-LINE1s are produced in both senses and have the same features as the canonical pathway sRNAs (Fig 7A and 7B). As expected, the lack of the canonical pathway (*dcl1 dcl2*Δ) provokes a decrease in sRNA production. Conversely, when the NCRIP is not active (*r3b2*Δ), there is an overaccumulation of antisense canonical sRNAs especially targeting the second open reading frame (ORF2) and its reverse transcriptase domain (RVT). The accumulation pattern and structural features of sRNAs from Grem-LINE1s were similar to those observed in a gene regulated by the canonical RNAi pathway (Fig 7). These results suggest an enhanced activity of the canonical RNAi machinery degrading the target retrotransposons when NCRIP is not active and therefore, a negative regulatory role for NCRIP. To explore the interaction among the two silencing pathways, the expression of the genes coding for key proteins of RNAi machinery was analyzed in the NCRIP mutants and in the wild-type strain during saprophytic conditions and phagocytosis (S5 Fig and S2 Data). Transcript levels of genes encoding Dcl1 and Dcl2 –the main ribonucleases in the canonical pathway–were increased in the NCRIP mutants in both conditions and also in the wild type during phagocytosis. This repression of the canonical pathway in the wild-type strain during saprophytic conditions suggests that NCRIP represses the canonical pathway under non-stressful stimuli.

## Discussion

Among the diversity of RNAi pathways in *M*. *circinelloides*, NCRIP is the most recently discovered. It is a new mechanism that remains mostly uncharacterized, and its functional role in fungal physiology is the central unanswered question [10]. Is it a non-canonical RNAi degradation mechanism that clears and turns over damaged RNAs? Or does it play a regulatory function controlling the expression of mRNAs at specific levels depending on cellular requirements? The results obtained in this study unveiled a complex regulatory role of NCRIP in fungal physiology rather than a simple degradation mechanism for functional or damaged RNAs. Thus, we identified thousands of genes regulated by NCRIP, including genes involved in survival during phagocytosis. The analysis of the spore response to the phagosome revealed a derepression of a complex gene network activated in the fungal spore after the interaction with macrophages. Moreover, we identified a negative regulatory role of NCRIP over the canonical RNAi pathway in the control of transposable elements, extending the functional complexity of this mechanism beyond the control of cellular mRNA levels. These intricate functional roles of NCRIP correlated with the pleiotropic phenotypes observed in mutants of this pathway [10], including the reduced virulence described here. Regarding the gene network regulated by NCRIP, previous studies suggested a broad regulatory function of this pathway based on the discovery of 611 loci producing sRNAs in a *dicer*-independent *rdrp*-dependent manner [10]. Here, we have directly analyzed the transcriptomic profiles in NCRIP key mutants, identifying a substantial number of DEGs in both *rdrp1Δ* and *r3b2Δ* mutants compared to the wild-type strain. However, a significantly lower number of genes were regulated in these mutants upon phagocytosis compared to the complex response observed in the wild-type strain [13]. The principal component analysis and the comparison of the four profiles among them further confirmed this strong bias among samples. Thus, NCRIP showed a differential regulatory intensity when saprophytic and phagocytic environments were compared. These results prompted us to hypothesize a repressive regulatory role of NCRIP under non-stressful conditions; hence, upon cellular challenges (like drug exposure or phagocytosis), the repression would cease allowing the activation of the corresponding gene response. Previous studies support this hypothesis, finding a similar regulatory mechanism in the epimutational pathway [3], which also suggested a negative regulatory role under non-stressful conditions [9]. A more in-depth analysis of the gene profiles and their expression levels supports our hypothesis because the mutants showed a pre-exposure adaptation to phagocytosis before the interaction with macrophages. This genetic response and the functions involved in it might explain the augmented oxidative stress tolerance observed in the NCRIP mutants *in vitro*. The pre-activated state of NCRIP mutants and their oxidative stress resistance suggest an advantage to resist the oxidative attack of macrophages. Surprisingly, the resistance to oxidative stress *in vitro* did not improve the pathogenicity of the mutants during *in vivo* interactions, shown as a reduced pathogenic potential in both *rdrp1*Δ and *r3b2*Δ mutants. These results reveal that NCRIP is necessary for virulence in Mucorales and could be explained because the intricate transcriptomic response displayed by the spores to counteract the host was not fully replicated in the NCRIP mutants. Indeed, we identified an NCRIP-dependent wild-type specific response, which is absent in the NCRIP mutants, that might explain the inability to develop the infection. This specific response is enriched in functions related to germination and iron transport and metabolism, which are essential for Mucorales to survive the host immunity [13,18,19]. Furthermore, the genetic deregulation in NCRIP mutants might affect other fungal responses required during the response to phagocytosis, or in further infection steps, such as tissue invasion, resulting in a final negative balance for the fungal spore.

Another regulatory role of NCRIP identified in this work unveiled a novel genetic mechanism in which the canonical RNAi pathway and NCRIP work as an antagonistic dual machinery to control the movement of transposable elements. Previous studies reported that most sRNAs produced by the NCRIP machinery map to exonic regions, but also to intergenic regions and transposable elements [10]. However, these studies were developed using initial annotation versions of the *M*. *circinelloides* genome before the identification of the centromeric regions. Once the centromeric regions were assembled, they were further characterized as rich in repetitive sequences and Grem-LINE1 retrotransposons [12]. The expression of the mobile elements is suppressed by the canonical RNAi machinery and correlates with an abundant production of sRNAs and low mRNA levels. In this work, we found an exacerbated production of antisense canonical sRNAs from centromeric retrotransposons in the NCRIP mutants, indicating an overactivation/derepression of the canonical RNAi pathway. Indeed, the NCRIP represses the expression of genes encoding key proteins of the canonical RNAi machinery. Altogether, these results suggest a negative regulatory role of NCRIP over the canonical RNAi pathway, analogous to the inhibitory role that the NCRIP pathway exerts over the epimutational pathway [3,9]. Via the canonical RNAi machinery, the epimutational pathway silences target genes to overcome growth inhibition caused by antifungal compounds and generates epimutant strains that are resistant to drugs [3,4]. Conversely, the inactivation of NCRIP leads to an overproduction of epimutant strains [9], suggesting either a competition between NCRIP and the epimutational pathway for the transcripts of the target gene or repression of NCRIP over the canonical mechanism. Here, we propose that the same mechanism is operating in the control of the transposition of the Grem-LINE1s. This hypothesis explains that the retrotransposons are suppressed by the canonical RNAi machinery when the NCRIP is inactive, manifested by enhanced production of sRNAs in *r3b2*Δ and *rdrp1*Δ mutants. Thus, the interaction between both RNAi pathways could be essential for centromere identity, genome stability and integrity.

The role of the RNAi machinery in protecting genome integrity against the movement of transposons is important during mating in several fungal models. In *C*. *neoformans*, the mechanism of sex-induced silencing (SIS) defends the genome against transposons during sexual development, whereas in several ascomycetes [20–22] an RNAi mechanism operates to silence unpaired DNA in meiosis (MSUD), including transposons [23]. These surveillance mechanisms that protect genome integrity rely on the RNAi canonical core, as in *M*. *circinelloides*. However, in this fungus, the canonical RNAi pathway coexists with a regulatory mechanism based on NCRIP, which has not been described in other fungal groups [10]. It is tempting to speculate that both the canonical mechanism and NCRIP perform a fine control over retrotransposable movements to gain genetic diversity in particular stressful conditions, allowing a transient activation of the retrotransposons to overcome the insult. Alteration of this precise control may be responsible for the defective mating observed in NCRIP mutants [10].

In conclusion, our functional study unveiled a complex gene network conditionally regulated by NCRIP. The analysis of this gene network revealed a remarkable function of NCRIP in the negative regulation of the genetic response elicited during phagocytosis, suggesting an essential role for this pathway in host-pathogen interactions. Altogether, the identification of a large number of genes regulated by NCRIP and the subset involved in the virulent response to phagocytosis confirm the broad regulatory role of NCRIP, arguing against a simpler role in clearance and turnover of RNAs. Instead, NCRIP emerges as a mechanism controlling an extensive network of genes involved in different cellular processes, with the capability of regulating them differentially after environmental challenges that include antifungals agents, phagocytosis, and virulence. The role of NCRIP controlling the genetic response to phagocytosis and the final phenotypic balance impairing virulence are new contributions to understanding the difficulty of treating and the challenge to manage the fungal infection mucormycosis.

## Materials and Methods

### Ethics statement

To guarantee the welfare of the animals and the ethics of any procedure related to animal experimentation, all the experiments performed in this work complied with the Guidelines of the European Union Council (Directive 2010/63/EU) and the Spanish RD 53/2013. Experiments and procedures were supervised and approved by the University of Murcia Animal Welfare and Ethics Committee and the Council of Water, Agriculture, Farming, Fishing and Environment of Murcia (Consejería de Agua, Agricultura, Ganadería, Pesca y Medio Ambiente de la CARM), Spain (authorization number REGA ES300305440012).

### Fungal strains, cell cultures, and RNA purification

The fungal strains used in this work derived from *M*. *circinelloides* f. *lusitanicus* CBS277.49. The wild-type control strain for the RNA-seq analysis and virulent control strain for the infection assays is R7B [24]. The strains defective in the NCRIP are MU419 (*rdrp1*Δ) [25] and MU412 (*r3b2*Δ) [10]. The strain R7B possesses the same auxotrophies and genetic background as the NCRIP mutants, except for their corresponding deletion in the *r3b2* or *rdrp1* genes (all the strains are auxotrophic for leucine due to a mutation in the *leuA* gene [26]). The strain NRRL3631 was used as an avirulent control for the mice infection experiments [27]. *M*. *circinelloides* cultures were grown in rich media YPG pH 4.5 at 26°C for optimal growth and sporulation. Spores were harvested and filtered using a Falcon 70 μm cell strainer before confronting with macrophages or animal models.

The host-pathogen interactions were performed confronting spores from R7B, MU419, and MU412 with mouse macrophages (cell line J774A.1; ATCC TIB-67) in a ratio 1.5:1 (spores:macrophages) following the protocol described in [13]. In summary, the interactions were maintained at 37°C in L15 medium (Capricorn Scientific) supplemented with 20% of Fetal Bovine Serum (FBS, Capricorn Scientific) for 5 hours, ensuring all the spores were phagocytosed. For saprophytic conditions, the same concentration of spores was cultured in L15 medium supplemented with 20% FBS but without macrophages.

For RNA purification, two replicates of each sample were pooled, and RNA was extracted using the RNeasy Plant Mini Kit (Qiagen, Hilden, Germany), following the manufacturer procedure.

Oxidative stress assays were performed in minimal media [28], either yeast nitrogen base (YNB) supplemented with leucine (20 mg/L) or minimal medium with casamino acids (MMC), both adjusted at pH 3.2. H_2_O_2_ was added to the media at two different concentrations: 5 mM and 10 mM, as well as control media without H_2_O_2_. 200 spores from each mutant and wild-type strain were plated in triplicates for each condition. Then, plates were grown at 26°C in the dark for 48 hours. Survival rate was computed as the ratio between growing colonies on H_2_O_2_-medium and control medium. Statistical differences among survival rates were assessed by a two-way ANOVA with Tukey’s multiple comparison test (95% confidence interval).

### RNA-sequencing analysis for gene expression and small RNA production

Raw datasets from *M*. *circinelloides* single- and co-cultures with mouse macrophages were quality-checked using FASTQC v0.11.8 before and after removing adapter and contaminant sequences with Trim Galore! v.0.6.2 (available at http://www.bioinformatics.babraham.ac.uk/projects/). Messenger RNA reads with a Phred quality score (Q) ≤ 32 and/or a total length ≤ 20 nt were removed from the analysis, as well as adapter sequences with an overlap ≥ 4 bases. These mRNA processed reads were aligned to the *M*. *circinelloides* f. *lusitanicus* v2.0 genome (herein Mucci2 [29], available at https://mycocosm.jgi.doe.gov/Mucci2/Mucci2.info.html) using STAR v.2.7.1a [30] and the subsequent Binary Alignment Maps (BAM) were used to create individual count matrices with HTSeq v.0.9.1 [31], excluding multi-mapping reads. Differential gene expression and principal components analysis (PCA) were performed by *limma* package v.3.38.3 [32] with robust settings for empirical Bayes statistics and the trimmed mean of M values (TMM) method for sample normalization; genes above a reliable threshold used in a previous study (False Discovery rate [FDR] ≤ 0.05; log_2_ fold change [log_2_ FC] ≥ 1.0; and average count per million reads (CPM) ≥ 1.0) [13] were considered differentially expressed genes (DEGs) and used for downstream analyses unless more stringent criteria is stated. Correlation of gene expression values (as log_2_ CPM) among samples analyzed for differential expression was assessed by examining scatter plots, highlighting up- and downregulated genes. In addition, three housekeeping genes whose expression is equal among samples were highlighted: translation elongation factor EF-1 (Mucci2 ID: 156959), RNA polymerase III transcription factor IIIC subunit TFIIIC (Mucci2 ID: 106349), and vacuolar-type H^+^-ATPase V-ATPase (Mucci2 ID: 154376). DEGs were clustered in heatmap plots using the pheatmap package v1.0.12 (available at https://cran.r-project.org/web/packages/pheatmap/) with Canberra distance and Ward’s clustering method. All genes were classified according to Eukaryotic Orthologous Groups (KOG) and Gene Ontology (GO) terms using EggNOG-mapper v2.0 [33,34]. Then, KOG class enrichment analyses were performed with KOGMWU package v1.2 [35]; delta-ranks were computed as the difference between the mean rank of all genes within the KOG class and the mean rank of all other genes in a Mann-Whitney U-test. A KOG class was considered over-represented if P ≤ 0.05 in a one-sided Fisher’s exact test evaluating the differentially expressed genes compared to the total amount of genes in each KOG class.

Both small and messenger RNA raw data employed to analyze the interaction between RNAi pathways targeting the centromeric retrotransposons were obtained from previous studies (see Data Availability). The mRNA raw data were processed as previously stated, but the reads were aligned to the *M*. *circinelloides* f. *lusitanicus* MU402 v1.0 genome (herein Muccir1_3 [12], available at https://mycocosm.jgi.doe.gov/Muccir1_3/Muccir1_3.info.html). This genome was PacBio-sequenced using long reads and thus, exhibit a greater content of repeated elements [12]. The sRNA raw data were processed with the aforementioned tools, excluding reads with Q ≤ 20 and/or total length ≤ 13 nt. Adapter sequences overlapping ≥ 2 bases at the 3’-end were removed. Processed sRNA were aligned to the Muccir1_3 genome using the Burrows-Wheeler Aligner (BWA) v.0.7.8 [36]. Aligned sRNA reads were split in those mapping to the forward and reverse strand by filtering through their Binary Alignment/Map (BAM) FLAG field (-F 16 for forward reads and -f 16 for reverse reads) with SAMtools v1.10–2 [37], to identify sense and antisense sRNAs. Forward or reverse reads aligning to same-sense protein-coding loci were considered sense sRNAs, and vice versa for those aligning to opposite-sense protein-coding loci (antisense sRNAs). Coverage was normalized to bins per million reads (BPM) in 25-bp bins with deepTools v3.2.1 [38] bamCoverage function. The resulting bigWig files were visualized with the deepTools pyGenomeTracks module using the centromeric and transposable element annotations found by Navarro-Mendoza et al. 2019 [12]. A differential sRNA production analysis across all protein-coding and Grem-LINE1 loci was conducted using *limma* with TMM normalization. The resulting sRNA production values were correlated in scatter plots, highlighting the Grem-LINE1s and the three housekeeping genes mentioned above, which have a stable sRNA production among samples [EF-1 (Muccir1_3 ID: 1382517), TFIIIC (Muccir1_3 ID: 1386549), V-ATPase (Muccir1_3 ID: 1377858)]. Sense and antisense sRNAs mapping to Grem-LINE1 3.01 (Muccir1_3 genomic coordinates: scaffold_4:2351462–2357388) and two control loci regulated by each RNAi pathway were searched for canonical and NCRIP sRNA features. The two control loci comprised a serine/threonine kinase controlled by the canonical RNAi pathway (Muccir1_3 ID: 1455000) and an alkaline phosphatase regulated by NCRIP (Muccir1_3 ID: 1469159). Base-conservation across these sRNA sequences was studied by generating nucleotide probability logos with WebLogo3 v3.7.4 [39]. In addition, size distribution probability of these sRNA was examined in a bar plot.

### RT-qPCR quantification

Replicate samples for the host-pathogen interactions and control conditions were used for RT-qPCR analysis. Once the mRNA was purified and treated with TURBO DNase (Thermo Fisher), the cDNA was synthesized from 1μg of total RNA using the iScript cDNA synthesis kit (Bio-Rad). The RT-qPCR was performed in triplicate using 2X SYBR green PCR master mix (Applied Biosystems) with a QuantStudio TM 5 flex system (Applied Biosystems) using 2X SYBR green PCR master mix (Applied Biosystems) following the supplier’s recommendations. To ensure non-specific amplification, non-template control and melting curve were tested. The primer sequences used for the quantification of genes *atf1*, *atf2*, *pps1*, *aqp1*, and rRNA *18S* are listed in S1 Table. The efficiencies of every pair of primers were approximately identical; thus, the relative gene expression of the target genes was obtained by the delta-delta cycle threshold (ΔΔCt) method, normalizing for the endogenous control rRNA *18S*.

### Virulence assays

The murine infection assays for Mucorales virulence were performed using OF-1 male mice weighing 30g (Charles River, Barcelona, Spain) [13,18,27]. The mice were immunosuppressed with the administration of cyclophosphamide (200 mg/kg of body weight) via intraperitoneal injection, 2 days prior to infection and once every 5 days thereafter. Groups of 10 mice were challenged with 1x10^6^ spores of the strains R7B, NRRL3631, MU419, and MU412. The infections were performed intravenously via retroorbital injection following the protocol described by Chang et al. 2019 [5]. Before the injection, mice were anesthetized by inhalation of isoflurane, and then the animals were visually monitored while recovering from the anesthesia. Mice were housed under established conditions with free food and autoclaved water. The animal welfare was checked twice daily for 20 days, and those following the criteria for discomfort were euthanized by CO_2_ inhalation. The significance of survival rates was quantified using the Kaplan-Meier estimator (GraphPad Prism). Differences were considered statistically significant at a P ≤ 0.05 in a Mantel-Cox test.

## Supporting information

S1 Data Differentially expressed genes in NCRIP mutants *r3b2*Δ and *rdrp1*Δ. (XLSX)

S2 Data Differentially expressed genes in NCRIP mutants analyzed as replicates. (XLSX)

S3 Data Differential sRNA production in genomic loci. (XLSX)

S1 Table Primers used in the study. (DOCX)

S1 Fig Correlation of expression values in *r3b2*Δ, *rdrp1*Δ mutant, and wild-type strains. Scatter plots of gene expression values (in log_2_ CPM, mean CPM > 0) in NCRIP mutants *r3b2*Δ and *rdrp1*Δ during saprophytic conditions (**A** and **B**, respectively) and during phagocytosis (**C** and **D**, respectively) compared to the wild type. Each dot shows the expression values of a gene, showing differentially upregulated genes (log_2_ FC ≥ 1.0, FDR ≤ 0.05) in red, and downregulated (log_2_ FC ≤ -1.0, FDR ≤ 0.05) in blue. Three housekeeping genes (coding for EF-1, TFIIIC, and V-ATPase) are shown in yellow to assure normalization among samples.
(TIF)

S2 Fig Correlation of expression values in NCRIP mutant and wild-type strains during phagocytosis. Scatter plots of gene expression values (in log_2_ CPM, mean CPM > 0) of the wild-type strain (**A**) and both NCRIP mutants analyzed as replicates during phagocytosis (**B**) and saprophytic growth (**C**). Each dot shows the expression values of a gene, showing differentially upregulated genes (log_2_ FC ≥ 1.0, FDR ≤ 0.05) in red, and downregulated (log_2_ FC ≤ -1.0, FDR ≤ 0.05) in blue. Three housekeeping genes (coding for EF-1, TFIIIC, and V-ATPase) are shown in yellow to assure normalization among samples.
(TIF)

S3 Fig Correlation of sRNA values in canonical and non-canonical RNAi mutants, and wild-type strain. Scatter plots of sRNA values (in log_2_ CPM, mean CPM > 0) of the canonical RNAi mutants *dcl1*Δ *dcl2*Δ (**A**) and *ago1*Δ (**B**); and NCRIP mutants *r3b2*Δ (**C**) and *rdrp1*Δ (**D**). Each dot shows sRNA values found in a given locus, showing the Grem-LINE1s in red, and three housekeeping loci (coding for EF-1, TFIIIC, and V-ATPase) in yellow to assure normalization among samples.
(TIF)

S4 Fig Transcript levels of transposable elements correlate with sRNA production. Transcript levels mapped to the Grem-LINE1s at centromere *CEN4* (the kinetochore biding region is shown as a blue rectangle) in the wild-type strain, a canonical pathway deficient strain (*dcl1*Δ *dcl2*Δ) and an NCRIP deficient mutant (*rdrp1*Δ) after 48 h of growth in rich medium. Transcript values are normalized to bins per million (BPM) mapped reads.
(TIF)

S5 Fig The expression of canonical RNAi pathway genes is affected by NCRIP activity. The expression values (calculated as the Z-score of CPM values) of all protein-coding genes involved in *M*. *circinelloides* RNAi pathways were plotted in a heatmap for the wild-type and the NCRIP mutant strains, both during phagocytosis (M) and saprophytic growth (C). Genes and experimental conditions are clustered by similarity of their expression values. Each analyzed gene corresponds to the following JGI Mucci2 gene IDs: *dcl2* (104153) and *ago1* (104161) code for the main Dicer-like and Argonaute-like ribonucleases involved in the canonical RNAi pathway, respectively; *dcl1* (104148) encodes a Dicer-like enzyme, partially redundant to Dcl2; *qip1* (110517) encodes an exonuclease involved in the canonical RNAi pathway; *rnhA* (143979) code for a DEAD-like helicase involved in the canonical and non-canonical RNAi pathways; *rdrp2* (195368) and *rdrp3* (159162) encode RNA-dependent RNA polymerases involved in RNAi; *ago2* (195366) and *ago3* (104163) code for putative Argonaute-like enzymes not involved in vegetative RNAi.
(TIF)

S1 Striking Image RNA sequencing of macrophage-pathogen interaction assays revealed a non-canonical RNAi pathway that controls gene expression during virulence processes and genome stability. (TIF)
