# Supplementary material for: A non-canonical RNAi pathway controls virulence and genome stability in Mucorales
Source: PLoS Genet. 2020 Jul 13;16(7):e1008611. doi: 10.1371/journal.pgen.1008611 (PMC7377519; doi:10.1371/journal.pgen.1008611)
Supplement: S1 Table — (DOCX) [file pgen.1008611.s004.docx]

**S1 Table. Primers used in the study.**

| **NAME** | **SEQUENCE** | **USE** |
| --- | --- | --- |
| atf1F4 | AATGAAATCCCCTCCCACTC | *atf1* RT-qPCR |
| atf1R4 | TTGTGGAGGTGTATCGACCA | *atf1* RT-qPCR |
| atf2F2 | GCAACAAGCGATAAGCAACA | *atf2* RT-qPCR |
| atf2R2 | ATGACGAAGGCACTTGATCC | *atf2* RT-qPCR |
| pps1F1 | CAGGCTCCTCCTCCTCTTCT | *pps1* RT-qPCR |
| pps1R1 | ATGAGGTGGCATGAAGGAAC | *pps1* RT-qPCR |
| aqp1F2 | GCGTGCTGTGTTTGACAGTT | *aqp1* RT-qPCR |
| aqp1R2 | AAAAGAACGAGCAGGGTTGA | *aqp1* RT-qPCR |
| 18sRNAF | CCGACTAGAGATTGGGCTTG | *rRNA* 18s RT-qPCR |
| 18sRNAR | TCTGGACCTGGTGAGTTTCC | *rRNA* 18s RT-qPCR |
