# Supplementary figures and images for: A non-canonical RNAi pathway controls virulence and genome stability in Mucorales
Source: PLoS Genet. 2020 Jul 13;16(7):e1008611. doi: 10.1371/journal.pgen.1008611 (PMC7377519; doi:10.1371/journal.pgen.1008611)

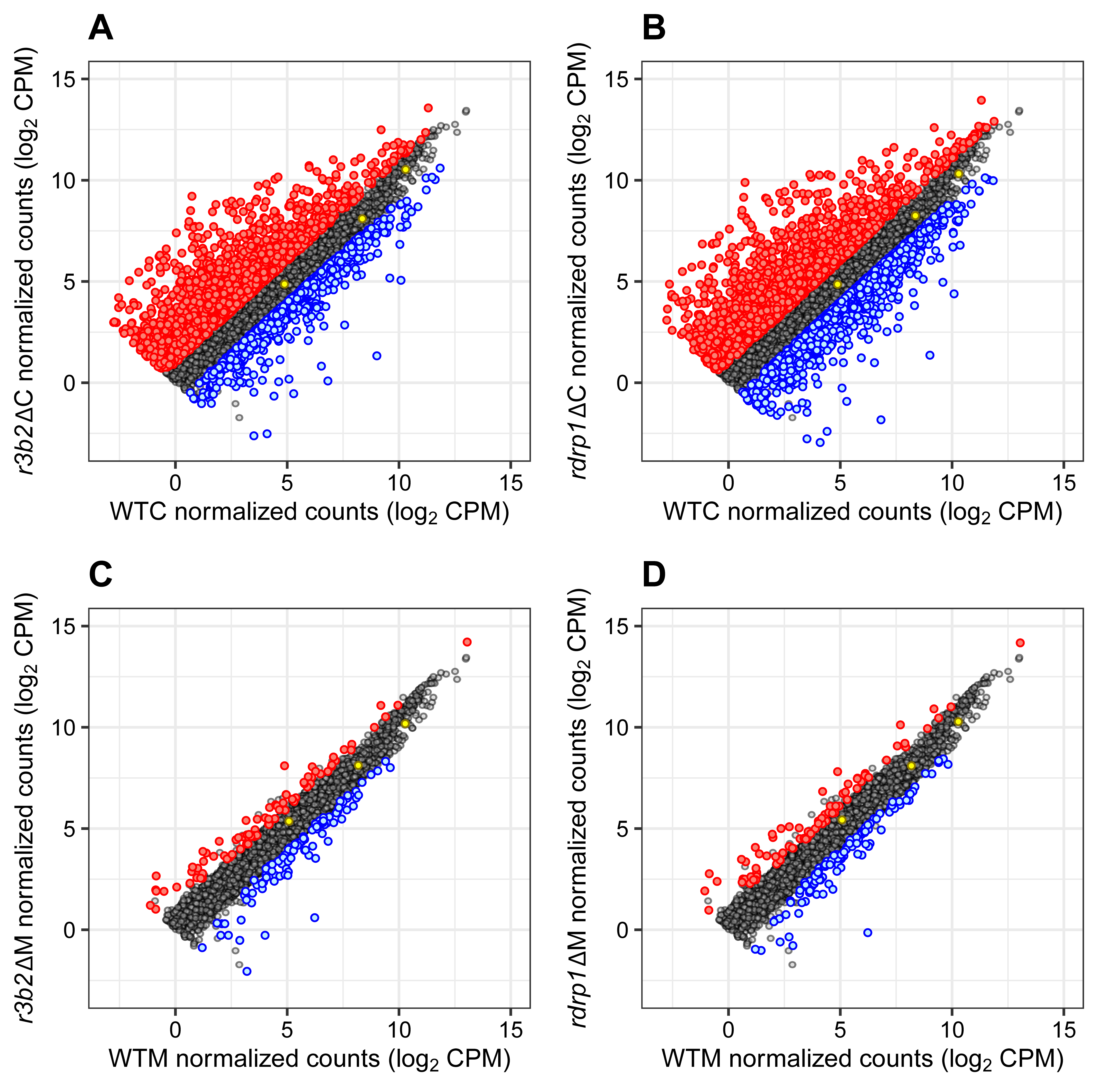

Supplement: S1 Fig — Scatter plots of gene expression values (in log2 CPM, mean CPM > 0) in NCRIP mutants r3b2Δ and rdrp1Δ during saprophytic conditions (A and B, respectively) and during phagocytosis (C and D, respectively) compared to the wild type. Each dot shows the expression values of a gene, showing differentially upregulated genes (log2 FC ≥ 1.0, FDR ≤ 0.05) in red, and downregulated (log2 FC ≤ -1.0, FDR ≤ 0.05) in blue. Three housekeeping genes (coding for EF-1, TFIIIC, and V-ATPase) are shown in yellow to assure normalization among samples. (TIF) [file pgen.1008611.s005.tif]

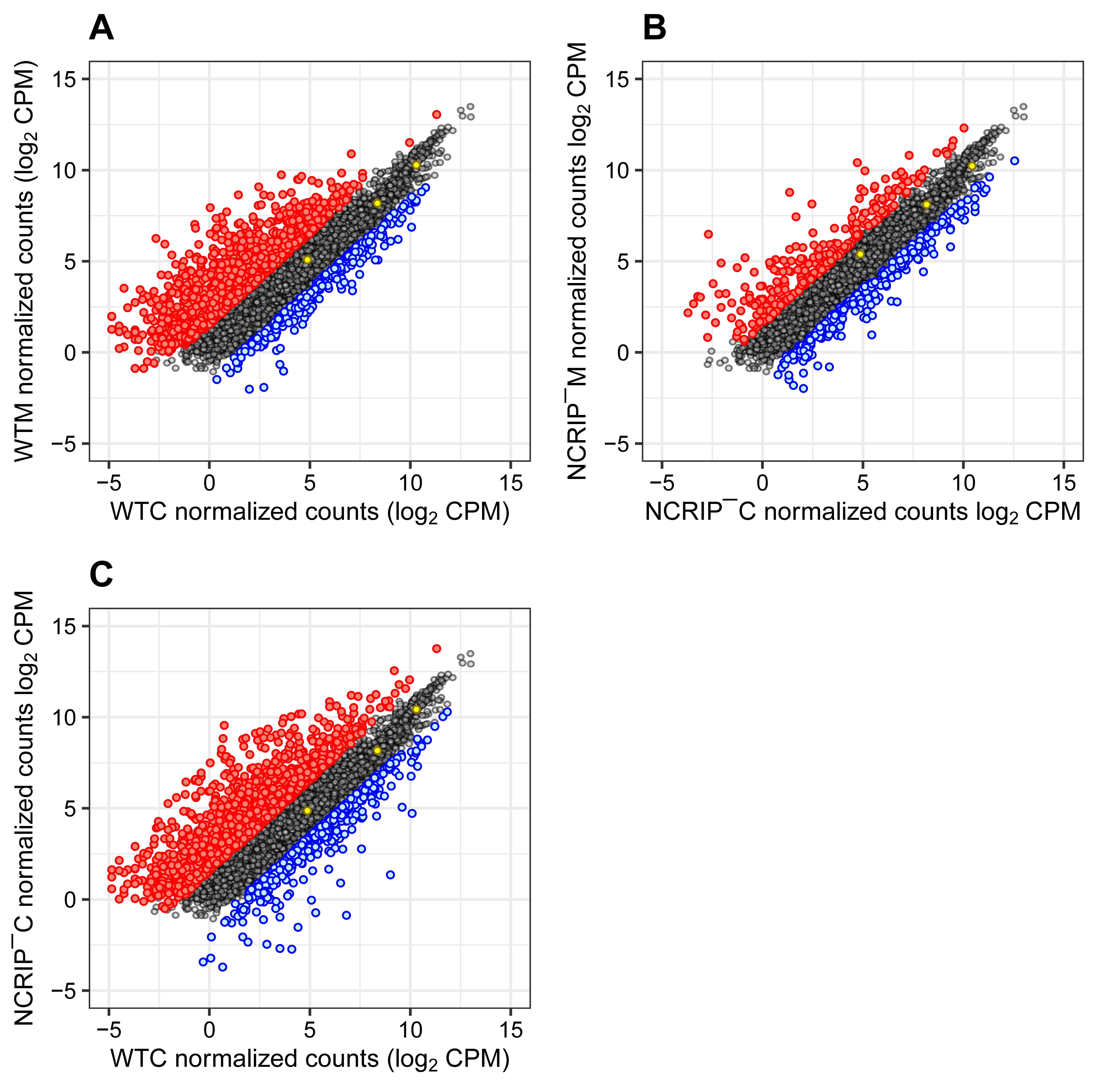

Supplement: S2 Fig — Scatter plots of gene expression values (in log2 CPM, mean CPM > 0) of the wild-type strain (A) and both NCRIP mutants analyzed as replicates during phagocytosis (B) and saprophytic growth (C). Each dot shows the expression values of a gene, showing differentially upregulated genes (log2 FC ≥ 1.0, FDR ≤ 0.05) in red, and downregulated (log2 FC ≤ -1.0, FDR ≤ 0.05) in blue. Three housekeeping genes (coding for EF-1, TFIIIC, and V-ATPase) are shown in yellow to assure normalization among samples. (TIF) [file pgen.1008611.s006.tif]

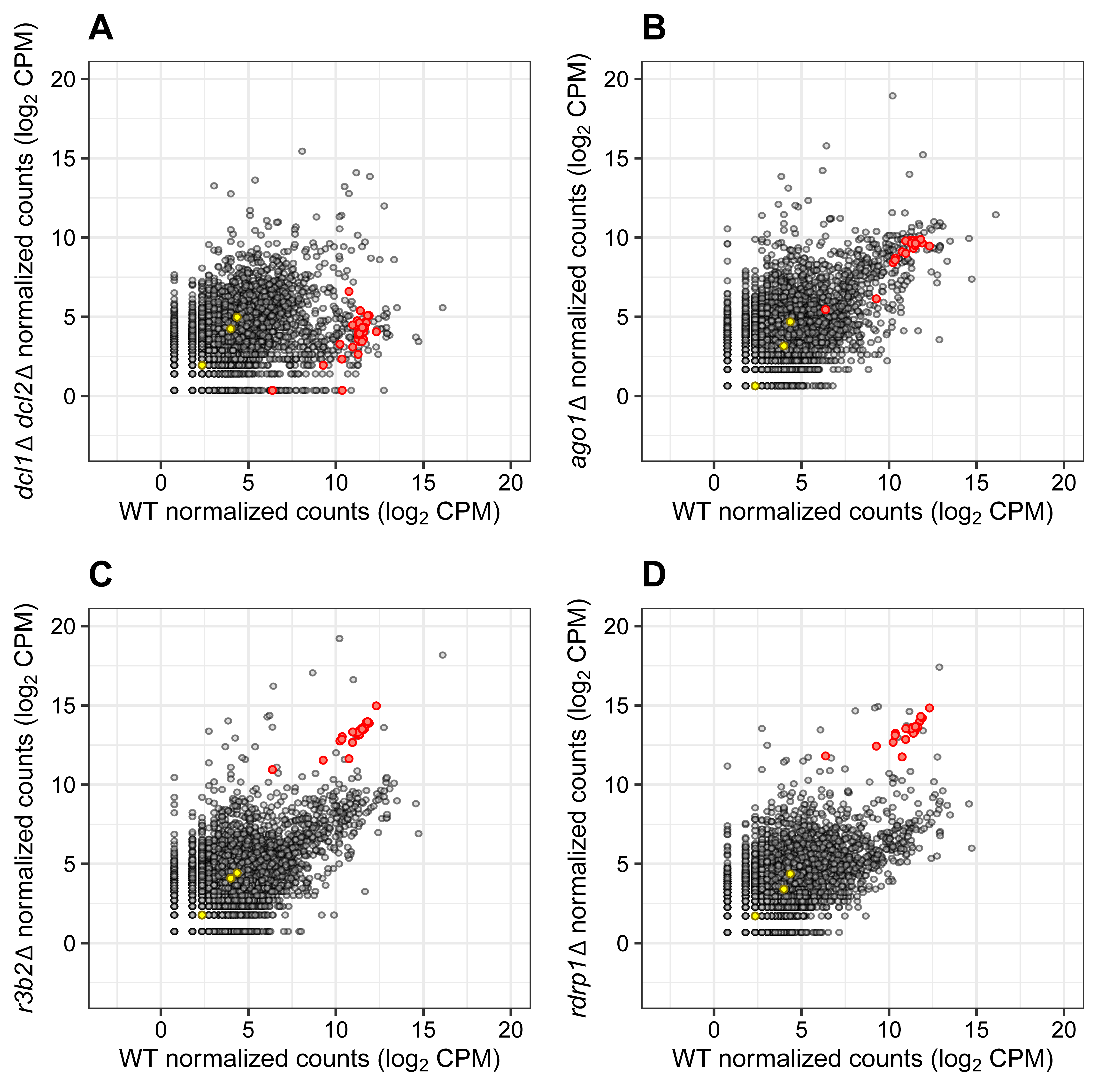

Supplement: S3 Fig — Scatter plots of sRNA values (in log2 CPM, mean CPM > 0) of the canonical RNAi mutants dcl1Δ dcl2Δ (A) and ago1Δ (B); and NCRIP mutants r3b2Δ (C) and rdrp1Δ (D). Each dot shows sRNA values found in a given locus, showing the Grem-LINE1s in red, and three housekeeping loci (coding for EF-1, TFIIIC, and V-ATPase) in yellow to assure normalization among samples. (TIF) [file pgen.1008611.s007.tif]

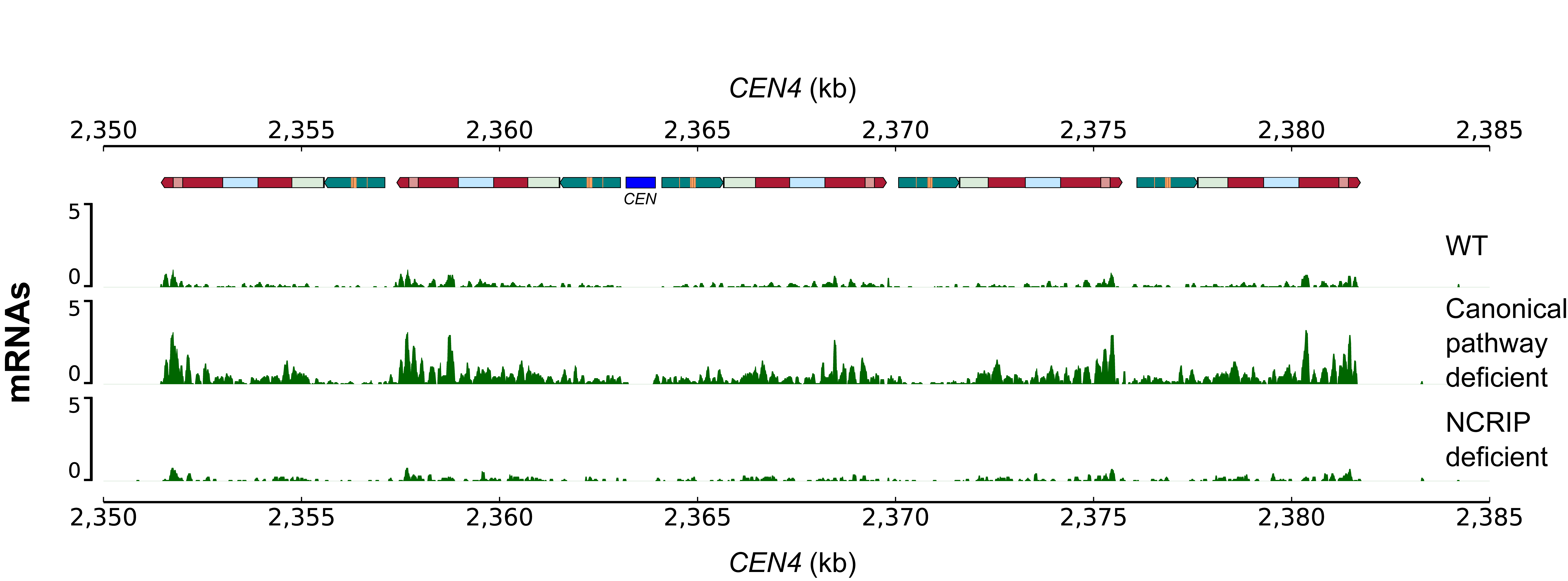

Supplement: S4 Fig — Transcript levels mapped to the Grem-LINE1s at centromere CEN4 (the kinetochore biding region is shown as a blue rectangle) in the wild-type strain, a canonical pathway deficient strain (dcl1Δ dcl2Δ) and an NCRIP deficient mutant (rdrp1Δ) after 48 h of growth in rich medium. Transcript values are normalized to bins per million (BPM) mapped reads. (TIF) [file pgen.1008611.s008.tif]

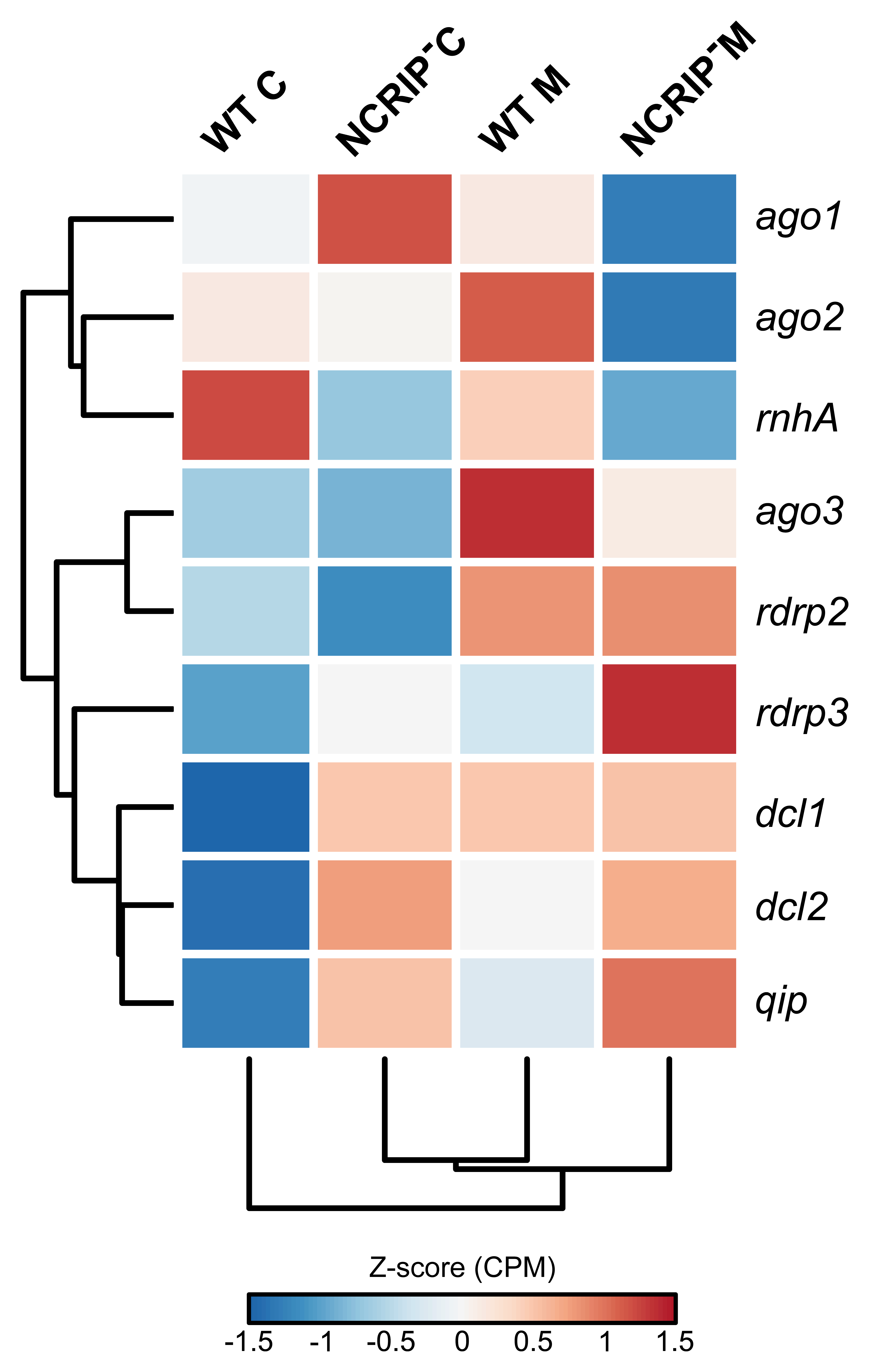

Supplement: S5 Fig — The expression values (calculated as the Z-score of CPM values) of all protein-coding genes involved in M. circinelloides RNAi pathways were plotted in a heatmap for the wild-type and the NCRIP mutant strains, both during phagocytosis (M) and saprophytic growth (C). Genes and experimental conditions are clustered by similarity of their expression values. Each analyzed gene corresponds to the following JGI Mucci2 gene IDs: dcl2 (104153) and ago1 (104161) code for the main Dicer-like and Argonaute-like ribonucleases involved in the canonical RNAi pathway, respectively; dcl1 (104148) encodes a Dicer-like enzyme, partially redundant to Dcl2; qip1 (110517) encodes an exonuclease involved in the canonical RNAi pathway; rnhA (143979) code for a DEAD-like helicase involved in the canonical and non-canonical RNAi pathways; rdrp2 (195368) and rdrp3 (159162) encode RNA-dependent RNA polymerases involved in RNAi; ago2 (195366) and ago3 (104163) code for putative Argonaute-like enzymes not involved in vegetative RNAi. (TIF) [file pgen.1008611.s009.tif]

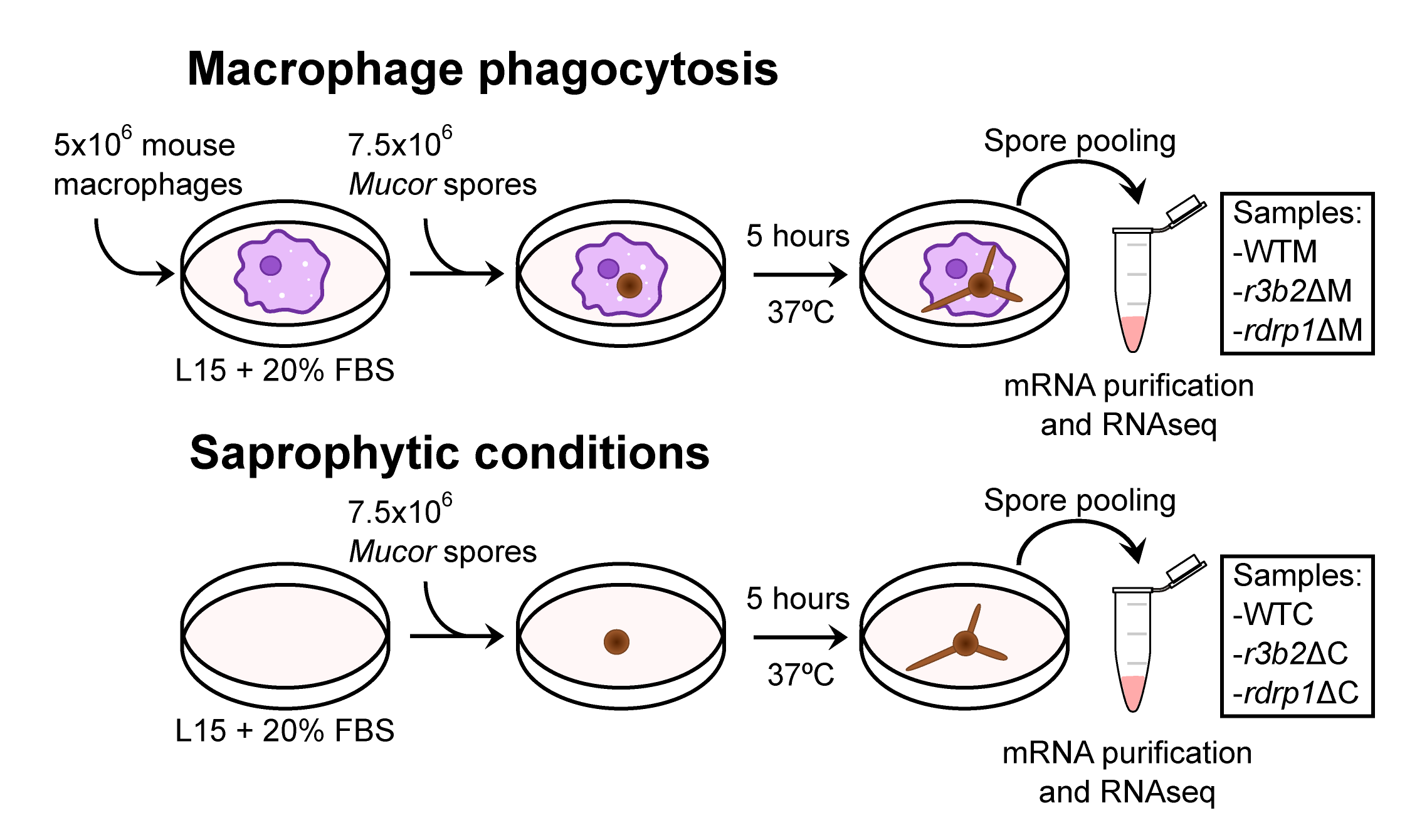

Supplement: S1 Striking Image — (TIF) [file pgen.1008611.s010.tif]
